# The Discrepancy between Coal Ash from Muffle, Circulating Fluidized Bed (CFB), and Pulverized Coal (PC) Furnaces, with a Focus on the Recovery of Iron and Rare Earth Elements

**DOI:** 10.3390/ma15238494

**Published:** 2022-11-29

**Authors:** Jinhe Pan, Xin Long, Lei Zhang, Andrei Shoppert, Dmitry Valeev, Changchun Zhou, Xiao Liu

**Affiliations:** 1 Key Laboratory of Coal Processing & Efficient Utilization, Ministry of Education, School of Chemical Engineering and Technology, China University of Mining & Technology, Xuzhou 221116, China; 2 Laboratory of Advanced Technologies in Non-Ferrous and Ferrous Metals Raw Materials Processing, Ural Federal University, 620002 Yekaterinburg, Russia; 3 Laboratory of Sorption Methods, Vernadsky Institute of Geochemistry and Analytical Chemistry, The Russian Academy of Sciences, 119991 Moscow, Russia; 4 Shenhua Zhungeer Energy and Resource Comprehensive Development Co., Ltd., Erdos 010300, China

**Keywords:** coal ash, circulating fluidized bed furnace, pulverized coal furnace, magnetic separation, rare earth elements, mode of occurrence, recovery

## Abstract

Coal ash (CA) is not only one of the most solid wastes from combustion, easily resulting in a series of concerns, but it is also an artificial deposit with considerable metals, such as iron and rare earth. The variation in the coal ash characteristics due to the origins, combustion process, and even storage environment has been hindering the metal utilization from coal ash. In this study, three ash sample from lab muffle, circulating fluidized bed (CFB), and pulverized coal (PC) furnace was derived for the discrepancy study from the combustion furnace, including properties, iron, and rare earth recovery. The origins of the coal feed samples have more of an effect on their properties than combustion furnaces. Magnetic separation is suitable for coal ash from PC because of the magnetite product, and the iron content is 58% in the Mag-1 fraction, with a yield of 3%. The particles in CA from CFB appear irregular and fragmental, while those from PC appear spherical with a smooth surface. The results of sequential chemical extraction and observation both indicated that the aluminosilicate phase plays an essential role in rare earth occurrences. Rare earth in CA from muffling and CFB is facilely leached, with a recovery of approximately 50%, which is higher than that from PC ash. This paper aims to offer a reference to easily understand the difference in metal recovery from coal ash.

## 1. Introduction

Coal ash (CA), as the product of coal combustion, is one of the main solid wastes in the world. Its presence with improper deposit has caused major concerns about the environment [1], economy [2], and human healthy [3]. The treatment or utilization of coal ash was subject to coal ash properties, resulting primarily from the coal source and combustion process. Generally, the feed materials of the coal power plant have complex compositions, which are the mixture of coal collected from different areas based on the calorific value [4]. Furthermore, the behavior of inorganic constituents in combustion furnaces or other procedures has a strong effect on the apparent properties of ash particles, such as size, roughness, and combination, which concerns the possibility and ability of physiochemical separation [5]. The coal combustion type is very important to coal ash utilization, especially critical materials recovery.

Coal combustion is a rapid chemical reaction between coal and oxygen, along with multiple complex reactions of other mineral matter. In the laboratory, a muffle furnace is a common faculty for the verification of coal combustion. In industry, coal is generally grilled and then injected into the furnace, which is called a pulverized coal (PC) furnace. This process could make coal sufficient for combustion with air and is applied in almost all thermal power plants. Unlike PC furnaces, circulating fluidized bed (CFB) boilers make coal stay in up-blow jets of air during the combustion, largely decreasing NO_X_ release and enhancing the in situ desulfurizing effect. Generally, the PC and CFB furnace temperatures are 1400–1500 °C and 850–900 °C [6], respectively, forming two different types of fly ash [7,8,9]. Hence, the combustion product from PC and CFB furnaces could be represented coal ash from the industry.

The properties of coal ash from these two furnaces are significantly different and profoundly influence the environmental treatment (e.g., metal leachability) and utilization (construction materials). The aluminum occurrence contains metakaolin and activated aluminum oxide in CFB ash, but it is mullite (Al_6_Si_2_O_13_) and corundum (α-Al_2_O_3_) in PC ash. Thus, direct high-pressure acid leaching adopts for Al recovery from CFB ash [10,11], and pretreatment before acid leaching [12] is necessary for Al recovery from PC ash. Similar phenomena happen on some heavy metals in both ashes. The concrete made by PC ash has better frost resistance properties than that made by CFB ash due to water absorption. The comparison between both ashes continues to be strengthened in view of the actuality of critical materials recovery from coal ash.

Coal ash, as an artificial metallic mineral deposit, is a promising resource of Al, Fe, Li, Ga, and so on [13,14]. Among various metals, rare earth elements recovery from coal ash is impressive due to the “Rare earth Program” by the Department of Energy, the U.S. It is reported that magnetic separation has a positive effect on Fe and rare earth enrichment [15,16]. Acid leaching is an important way to Al and rare earth recovery [17]. From previous reports, a contradiction always appears that the same or similar acid leaching conditions result in extremely different metal recovery from different coal ashes. Except for the origins of feed coal, the combustion process has absolutely necessary effects on those results.

In this study, three coal ash samples from lab muffle pulverized coal and circulating fluidized bed furnaces were studied to compare their properties containing density, specific surface area, particle size, petrology, and mineralogy. Magnetic separation was used for the Fe enrichment. The leaching experiments using mineral and organic acids were performed to evaluate the recovery of Al and rare earth from three ashes. This paper aims to reveal the difference in metal (Fe, Al, and rare earth elements) recovery from muffling ash, PC ash, and CFB ash.

## 2. Materials and Methods

### 2.1. Materials

In this paper, a coal gangue (CG) sample was collected from a coal preparation plant located in the Jungar coalfield of Inner Mongolia, where the coal enriches rare earth and yttrium (REY) [18]. The combustion product by the muffle furnace using the coal gangue as the feed was named coal ash No. 1 (CA1). The combustion condition is shown in detail in Section 2.2. Generally, valuable metal elements are more enriched in fly ash than bottom ash [19]. Hence, the fly ash from PC and CFB furnaces was studied in this research. The second coal fly ash (CA2) was taken from a CFB power generation plant, of which the feed is the CG sample mentioned. Another coal fly ash (CA3) was combusted by pulverized coal furnace at the Faer power plant in Guizhou Province.

### 2.2. Methods

The coal gangue sample was combusted for CA1, followed by the Chinese standard GB/T 34231-2017 [20]. The combustion process was set as five steps: rise up to T = 500 ± 10 °C for 30 min with a hold time at this temperature for 30 min, and then rise up to T = 815 ± 10 °C, with a hold time for 30 min. The last step is two inspections until constant weight. The repeated trials were performed for the degree of accuracy. The other two samples are also treated for loss on ignition (LOI).

A magnetic tube tester (or Davis tube tester) was used to investigate the distribution of iron and REY in the magnetism-fraction of three samples. The magnetic field strength was successively adjusted due to the change of the electric current (1A, 2A, 3A, 4A, and 5A), and the sample was defined as magnetic fractions (Mag-1, Mag-2, Mag-3, Mag-4, and Mag-5, correspondingly) and a non-magnetic fraction.

Sequential chemical extraction procedures (SCEP) were regarded as a significant method for the quantification of trace element occurrence. According to the reported method [21], REY occurrence was segmented into five fractions: (1) ion-exchangeable, (2) acid soluble, (3) metal oxides, (4) organic or sulfide, and (5) aluminosilicate.

Acid leaching is a process for metal extraction from primary and secondary resources and is also used for the identification of metal leaching ability at specific conditions. The REY leaching ability was defined by hydrochloric acid and citric acid (as a proxy for mineral and organic acid leaching) under measurable conditions based on the reported literature [22,23,24]. The hydrochloride acid leaching was performed in the beaker with the conditions: T = 60 °C, the concentration of 3 M, the liquid–solid ratio of 10 (volume/mass, *v*/*m*), leaching time of 120 min, and magnetic stirring speed of 200 rpm. The conditions of citric acid leaching contain a temperature of 90 °C, a concentration of 1 M, a liquid–solid ratio of 25 (volume/mass, *v*/*m*), a leaching time of 120 min, and a magnetic stirring speed of 200 rpm. The difference between the two leaching experiments is probably because of their properties, including the formula, ionization, and viscosity of the solution.

### 2.3. Analysis

X-ray fluorescence (XRF, S8 Tiger, Bruker AXS Corporation, China) was used to analyze the major element compositions in the CA samples. The trace elements in the leachate were measured using inductively coupled plasma mass spectroscopy (ICP-MS, Thermo ICAP RQ, Bremen, German). Before elemental analysis, the solid samples were digested in a microwave instrument using the method described in our previous study [25]. A scanning electron microscope (FE-SEM, ZEISS ΣIGMA, Oberkochen, German), in conjunction with an energy-dispersive X-ray spectrometer (Oxford X-MaxN 20, Shanghai, China) (collectively, SEM-EDS), was applied to observe the particles of iron and REY. The images were captured through a retractable solid-state backscatter electron detector, which was more easily to find REY/iron-containing minerals in view of the bottom position of the periodic table of elements. The specific surface area of CA samples was measured by an Autosorb-iQ analyzer (Quantachrome, Boynton Beach, FA, USA) followed by the BET method. The pycnometer method was used for the density determination of the samples by reference to distilled water. The particle size distribution of three samples was obtained using a laser diffraction analyzer (Microtract S3500, York, PA, USA).

## 3. Results and Discussion

### 3.1. Property Difference of Coal Ash Samples

#### 3.1.1. Chemical Composition

**Figure 1:**
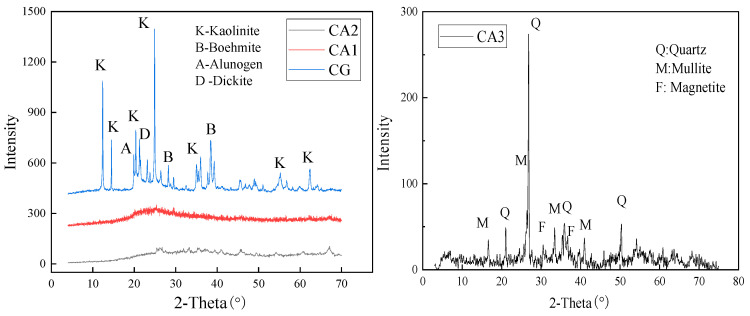
XRD patterns of three coal ash samples and coal gangue.

The XRD patterns of the three ash samples are drawn in Figure 1, along with the pattern of coal gangues as the feed materials of CA1 and CA2. It indicates that the main phases in coal gangue are kaolinite (Al_2_(Si_2_O_5_)(OH)_4_), boehmite (AlOOH), alunogen (Al_2_(SO_4_)_3_·17H_2_O), and dickite (Al_2_(Si_2_O_5_)(OH)_4_) and the peak of quartz (SiO_2_), as the most common phase in coal gangue (CG), does not appear, possibly because of the very small content. As shown in the XRD pattern, there is no peak of the mineral phase in the CA1 and CA2 patterns. The hump at the 2θ of 20~30° indicates the fact that the aluminosilicate minerals have been transferred into the amorphous matter, and the new crystal phase does not generate in the temperature range of 800–900 °C, consistent with the previous research [26,27]. It was shown that mullite and corundum would generate at higher temperatures, such as 1000–1200 °C, and was also reported in PC coal fly ash using the feed material from the Jungar deposits [28,29,30]. Additionally, CA3 is a typical F fly ash containing quartz, mullite, and magnetite.

The chemical composition of the three ash samples was analyzed by XRF and ICP-MS for major elements and rare earth elements plus yttrium (REY), as listed in Table 1. The ratio of Al/Si in CA1 is similar to that in CG based on the aluminosilicate minerals. The ratio of Al/Si in CA2 is vastly different from that in CA1 and CG. Except for the influence of the production batch, the quartz produced from kaolinite when burning probably was left in the bottom of the furnace as a loose mixture. CA3 is a high-silicate coal fly ash with moderate content of iron oxides. The existence of phosphorus implied the possibility of rare earth enrichment in coal-based resources, given their affinity [31].

The content of rare earth and yttrium (REY) in the three ash samples are 240.20 μg/g, 516.80 μg/g, and 520.27 μg/g, respectively. The outlook coefficient (C_outl_) is the ratio of the relative amount of critical REY (Nd, Eu, Tb, Dy, Er, Y) in the REY sum to the relative amount of excessive REY (Ce, Ho, Tm, Yb, Lu), which represents the potential industrial value [32]. The C_outl_ in three samples are 0.97, 0.86, and 0.88, indicating that the amount of critical and excessive REY is almost equal.

#### 3.1.2. Physical Properties

**Figure 2:**
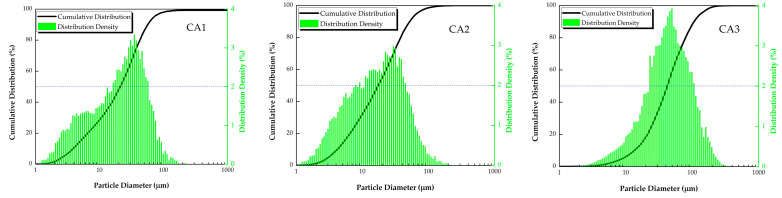
Particle size distribution of three coal ash samples.

As shown in Table 2, the physical properties of three coal ash samples, including density, specific surface area, LOI, and D50, were investigated. The density values indicated no significant difference in compositional homogeneity. Because CA1 inherits all inorganic constituents from coal, fly ash is only the part in suspension during the combustion. Another reason is thorough burning in the lab (without unburnt carbon from the data of LOI). The product of a hollow glass microsphere in the PC furnace possibly results in lower density. The proportion of iron in CA3 is notably higher than in the other two ashes. The results of density are as follow in Table 2. Not unexpectedly, CA3 has the largest specific surface area despite the glass microsphere generated with the higher temperature at the PC furnace than the CFB furnace. Analogously, the D50 of CA3 is also the highest among the three samples. The measured specific surface areas of CA2 are slightly higher than that of CA1 in view of organic matter, generally resulting in lower density and higher specific surface area. The D50 of CA1 and CA2 are similar probably because the synthesis of the organic matter (LOI: 4.11%) existed in CA2 and the fact that mineral matter in CA2 is thinner than that in CA1 due to the motion in the CFB furnace. Figure 2 shows the distribution of particle size of three ash samples. CA1 and CA2 have moderate proportions of the fractions of < 10μm, while the particles of CA3 are concentrated in the fractions of 20–70 μm. This difference was controlled by the feed materials (e.g., fragile aluminosilicate minerals) and combustion process (e.g., the glass phase production).

### 3.2. Magnetic Separation

**Figure 3:**
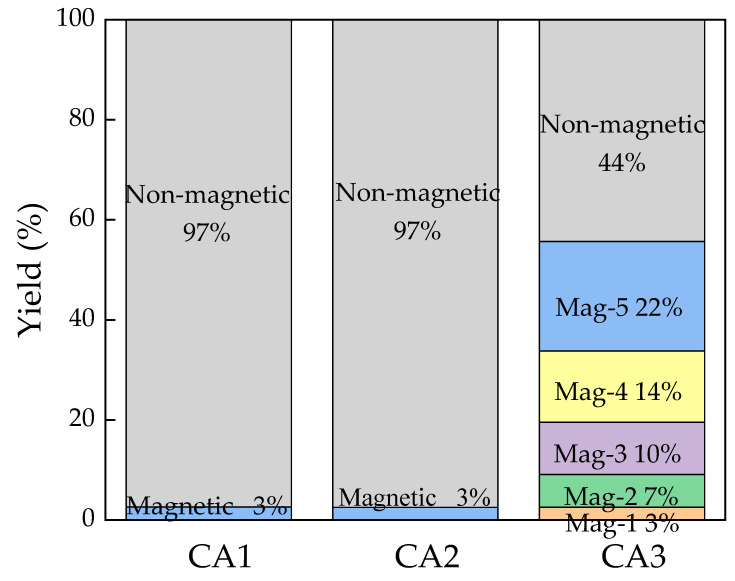
Magnetic fractions of three coal ash samples.

The magnetic separation results of the three samples are shown in Figure 3. Given the iron contents and the test phenomenon, all magnetic fractions of CA1 and CA2 were represented by the results of a magnetic separation experiment with 5A and accounted for 2.61% and 2.51%. Those results are consistent with the fact that iron oxide or hematite in fly ash was the dominant occurrence of iron when combusted at 800–900 °C [33]. On the contrary, CA3 was divided into six fractions by magnetic separation with different magnetic field intensities. One of the main reasons is that the dominant occurrence of iron in CA3 is the magnetite phase, which is easily captured at a weak magnetic field [34,35]. The combination of magnetite and other phases (e.g., glassy phase), and even the entrainment during the magnetic separation, led to a moderate proportion of magnetic fractions, including Mag-5 (22%), Mag-4 (14%), Mag-3 (10%), Mag-2 (7%), and Mag-1 (3%).

**Figure 4:**
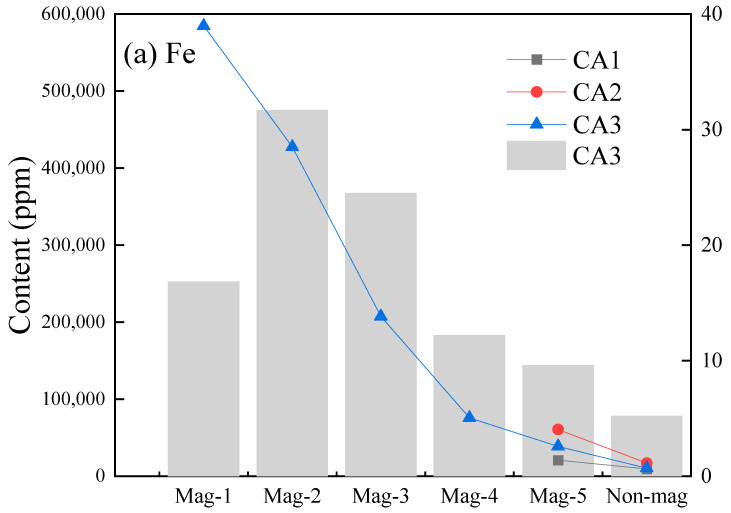

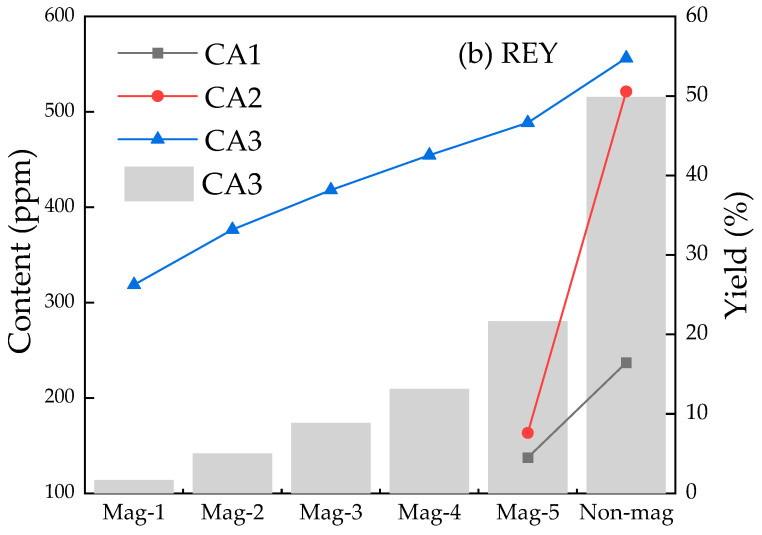
The contents and distribution of iron and REY in three coal ash samples.

All fractions of the three samples were digested for the contents of iron and rare earth by ICP-MS measurement. The distribution of iron and rare earth in CA1 and CA2 were not discussed here because the conclusions were clear at a glance due to the proportion of 3% and almost unchanged contents in non-magnetic fraction (97%) with raw samples. As Figure 4a shows, it is expected that the iron content decreases from Mag-1 to a Non-magnetic fraction. In the Mag-1 fraction, with a distribution of 17%, the content of iron is around 58% which reaches or closes to the lowest industrial grade [36]. The Mag-2 fraction, with an iron content of up to 42%, has the proportion iron (32%) of all in CA3. Both fractions account for nearly 50% iron of all in CA3 with an iron content of 47%, the weighted average gotten by the calculation. Thus, the iron recovery from coal ash will be profitable under certain conditions. Diversely, CA1 and CA2 contain a bit of iron, and even the 3% of iron contents in their magnetic fractions are distributed bilaterally to the Mag-5 point.

The REY contents in all fractions of CA3 increased from Mag-1 to non-magnetic fractions at opposite poles of that of iron contents from Figure 4b. The majority (50%) of REY was distributed in the non-magnetic fraction in which the REY content is 556 μg/g, a bit over that in the raw CA3 sample. Similarly, the non-magnetic fractions in CA1 and CA2 contain most REY, and the REY contents are slightly higher than that in the raw sample, respectively. The REY content in the magnetic fraction of CA2 is significantly less than that in the non-magnetic fraction, indicating the uncorrelation between REY and iron. Additionally, the analogical things appeared in CA1. In view of the small number of magnetic yields, magnetic separation is hard to be an efficient method for REY enrichment.

**Figure 5:**
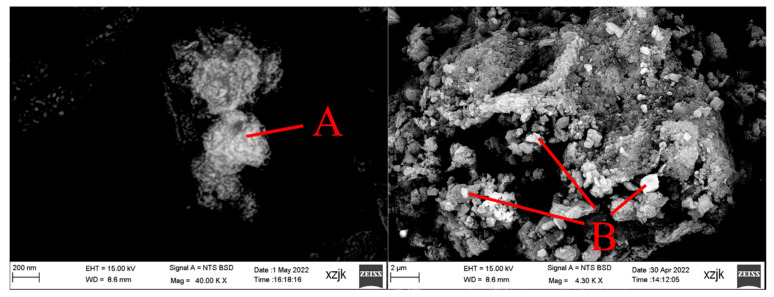

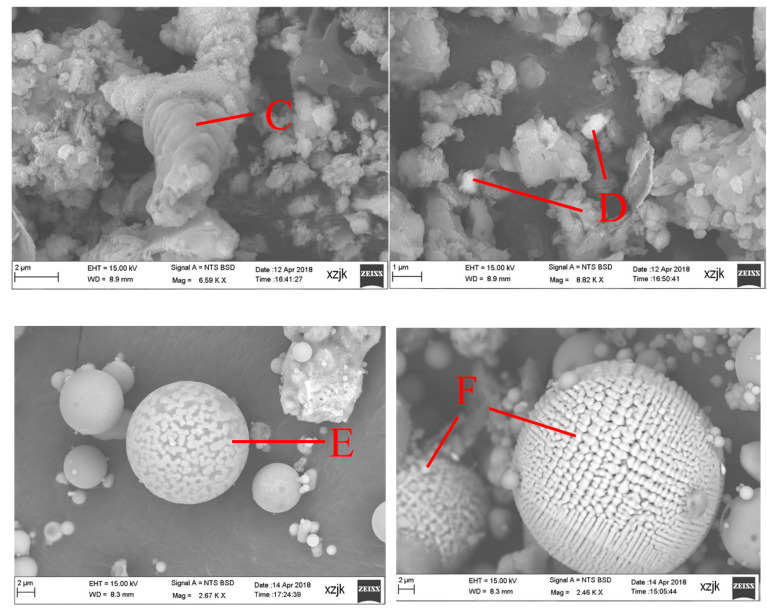
Results of SEM-EDS analysis for CA1 (**A**,**B**), CA2 (**C**,**D**), and CA3 (**E**,**F**).

The magnetic fraction in CA1 and CA2 and Mag-1 fraction in CA3 were observed by SEM-EDS, as shown in Figure 5 and Table 3. Point A is a typical hematite particle bond to the aluminosilicate phase. Point B indicated that the micro- or nano-particles of iron distribute on or surrounding the aluminosilicate phase. Point C is the heated kaolinite surrounding/reacting with other elements phase based on the stratified structure and elemental analysis. Point B is a magnetic particle coupled with the aluminosilicate phase. As the product of the CFB furnace, other areas except for points C and D represent the typical characteristic that the particles are irregular, fragmental, and agglomerated into large particles [36,37]. That is the reason why some particles were entrained into the magnetic fraction during the separation. Points E and F in CA3 are magnetic spheres inferred as the combustion product of pyrite framboid [38]. During the combustion, pyrite framboid is combined with molten peripheral or concomitant aluminosilicate minerals or glassy phases, leading to a glazed sphere wrapping the iron particles. While pyrite framboid did not get or get less glassy phases, the particles are coarse or stay intact, though oxidized.

### 3.3. Recovery of Rare Earth

#### 3.3.1. Modes of Occurrence of Rare Earth

**Figure 6:**
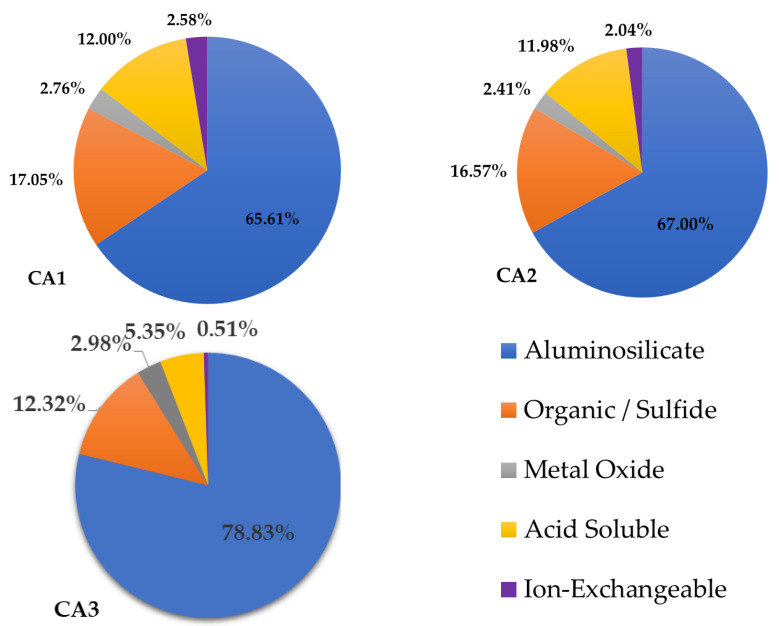
Modes of occurrence of rare earth elements in three samples.

The sequential chemical extraction procedure was used to quantitatively study the occurrence modes of rare earth and also reveal the leachability of rare earth in a mild environment. The results of the three coal ash samples are shown in Figure 6. As reported in previous research [31,39], the aluminosilicate fraction is the dominant mode of rare earth occurrence, accounting for 66%, 70%, and 79%, respectively. In other words, the amount of reserved rare earth in the solid residue keeps an order: CA3 > CA2 > CA1. It conforms to the order of temperature, and the existence of the glassy phase in CA3 sets the reserved rare earth proportion apart from those of the other two samples. The second mode of occurrence is the organic/sulfide phase, with a proportion of 17%, 17%, and 12%, respectively. The contents of carbon and sulfur in these three samples are not highlighted. It was referred that rare earth enriched in unburnt carbon and sulfate phase or rare earth was leached by the higher intensity of the solution than those of the front three solutions, which is also an adverse factor or system error of sequential chemical extraction procedure. Although some researchers found rare earth associated with the iron phase in coal fly ash using observation methods, the proportions of rare earth in metal oxide are only 2.76%, 2.41%, and 2.98%, respectively. Rare earth bonds to acid-soluble could be leached by weak acids in nature, and it is generally thought that rare earth is associated with carbonate minerals in coal. A small number of rare earths occurred in the ion-exchangeable fraction of these three samples. It is a positive deduction that around 30% of REY in CA1/CA2 and 20% of REY in CA3 could be directly leached under mild conditions.

#### 3.3.2. Rare Earth Carriers

**Figure 7:**
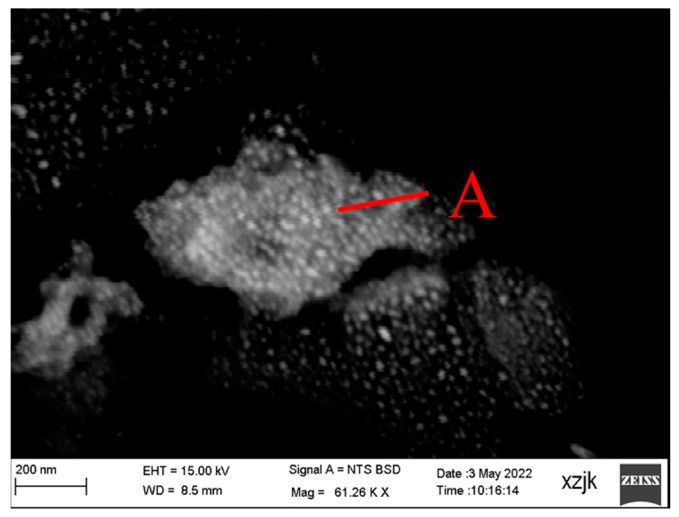

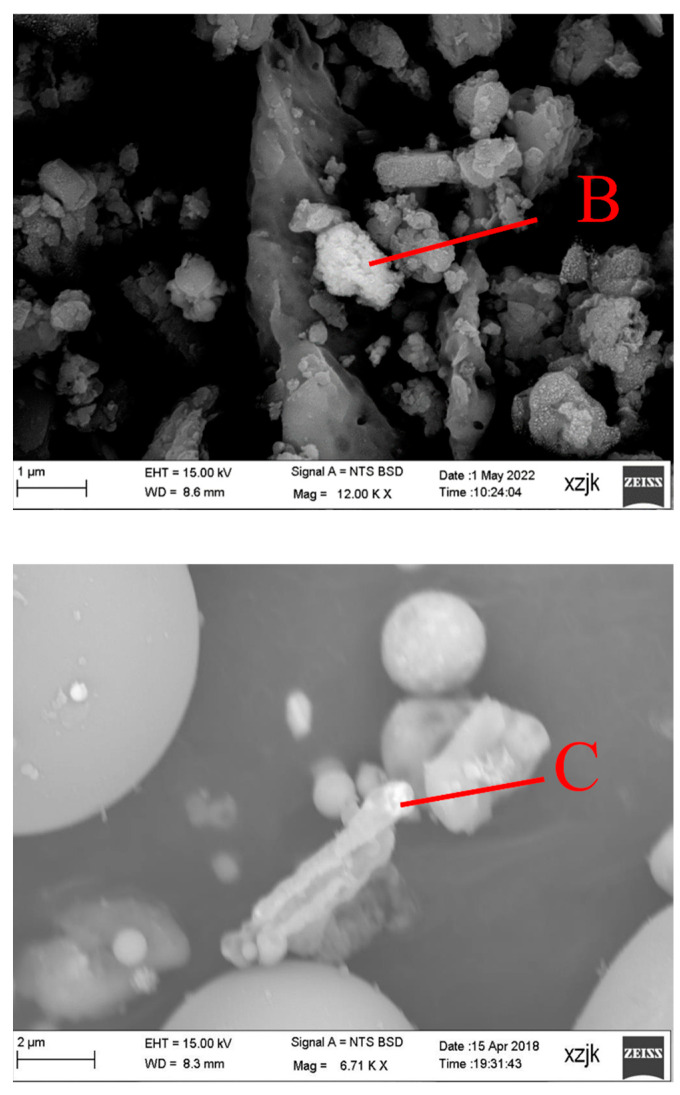
Rare earth carriers in CA1 (**A**), CA1 (**B**), and CA1 (**C**).

The carriers of rare earth in coal-based resources contain phosphate minerals, iron oxide, clay minerals, and carbon, as in the reported literature [40,41,42,43,44]. The results of SEM-EDS analysis for three coal ash samples are shown in Figure 7 and Table 4. Rare earth carriers are bright areas or particles. Point A in CA1 and Point B in CA2 are inferred to be monazite and apatite as nano-particles based on morphology and elemental composition. Those nano-particles almost strewed on the aluminosilicate phase with a small amount of iron and titanium or independently scattered around Point A, which is the product of muffle or static combustion. Based on the element analysis, there is no phosphorus in Point C, in which rare earth account for 25% of wt. It has been indicated that Point C is from rare earth bonds to clay minerals in raw coal. At the complicated condition during the combustion, it is hard for rare earth carriers to be dissociated well with other phases so that physical separation does not fit rare earth enrichment in coal ash. Given the mentioned fact, the importance of the hydrometallurgical method stands out for rare earth recovery.

#### 3.3.3. Acid Leaching

**Figure 8:**
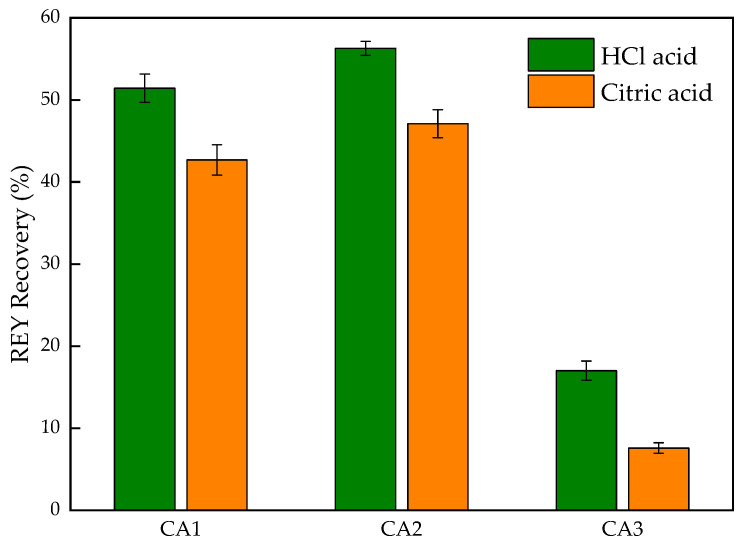
Rare earth elements in three coal ash samples.

Acid leaching is an efficient, economical, and widely used process in the rare metal metallurgical industry and has an absolutely necessary role in rare earth recovery from coal fly ash. Hence, the leachability of rare earth in the three samples was evaluated using mineral acid (HCl acid) and organic acid (citric acid) at the specific conditions verified in previous research [22,23,24,45]. On the whole, the recovery of REY using HCl acid is higher than that using citric acid from Figure 8. Because the amount of hydrogen ions in citric acid solution is less than that in HCl acid solution, it resulted in the weak ability of dissolution. Meanwhile, other metals, such as Al and Fe, should be leached more easily by HCl acid than citric acid based on acidity, although the data of other elements are not shown here. Organic acid or a mix of the organic acid may be an efficient design in acid leaching for REY recovery, which brings the organo-functional group advantages to the full. Furthermore, the leaching selectivity for REY and other major elements in coal ash is expected based on the recent literature [20]. As about mentioned, CA1 and CA2 have similar properties, leading to similar REY recovery, though the REY recovery in CA2 is slightly higher than that in CA1, possibly because of the particle size. The REY recovery in CA3 is significantly less than that in other samples due to the existence of glassy phase, wrapping, and bounding REY carriers. The ash from the CFB furnace is more conducive for REY recovery via acid leaching, which does not need pretreatment for the broken glassy phase. Another advantage is to avoid the impurity from glassy phase dissolution during the downstream, such as solvent extraction and resin sorption.

## 4. Conclusions

In this research, the discrepancy of the three coal ash samples from a lab muffle furnace, circulating fluidized bed, and pulverized coal furnace was studied from the aspects of properties and recovery of iron and rare earth. CA1 and CA2 contain high aluminum content and low iron content resulting from the geologic feature of feed materials. CA3 has a moderate content of iron existence in magnetite. The phase peak in the XRD pattern of CFB ash is not more obvious than that of PC ash due to the uncompleted transformation of aluminosilicate. Although a clear trend of physical property differences can not be concluded based on a case study, the unburnt carbon and glassy phase play very important roles in the value of the individual physical property.

The magnetic fractions in CA1 and CA2 account for less than 3%, while that in CA3 accounts for over 50%. The iron content in Mag-1 of CA3 is nearly 60%, with a yield of 3%. The majority of REY was distributed in non-magnetic fractions with little enrichment than that in the raw sample. It was found in CA1 and CA2 magnetic fractions that the iron particles with micro size were embedded in or bonded to the aluminosilicate phase. The product of pyrite framboid was observed in the CA3 Mag-1 fraction, appearing as independent spheres.

The content of iron in CA1, CA2, and CA3, are 240.20 μg/g, 516.80 μg/g, and 520.27 μg/g, respectively, with positive outlook coefficients. REY bond to the aluminosilicate phase in CA3 is higher than that in CA1 and CA2, indicating a strong leachability of REY in CA1 and CA2. The leaching results support the conclusion that CFB ash has a better leachability for REY than PC ash. The carriers of REY in three samples are all found via SEM-EDS analysis. REY, in this study, has an affinity with phosphorus, iron, and titanium.

## Figures and Tables

**Table 1:** Chemical composition of three coal ash samples (%, μg/g).

Elements	CA1	CA2	CA3	Elements	CA1	CA2	CA3
SiO_2_	44.15	25.19	52.17	La	46.73	88.64	80.65
Al_2_O_3_	46.83	57.37	23.89	Ce	77.50	191.61	193.19
Fe_2_O_3_	1.35	2.587	12.1	Pr	10.63	21.98	22.47
CaO	1.56	2.84	2.36	Nd	34.62	89.64	88.66
MgO	0.07	0.16	1.91	Sm	6.67	16.92	17.22
TiO_2_	0.96	2.07	2.77	Y	37.40	57.93	65.17
K_2_O	0.20	0.35	0.69	Eu	1.11	3.84	3.33
Na_2_O	0.23	0.67	0.58	Gd	6.06	14.89	15.53
S	0.19	0.31	0.22	Tb	1.15	2.18	2.20
P	0.07	0.10	0.14	Dy	7.38	12.71	13.60
				Ho	1.45	2.40	2.58
				Er	4.37	6.58	7.17
				Tm	0.62	0.92	1.01
REY	240.20	516.80	520.27	Yb	3.95	5.73	6.57
C_outl_	0.97	0.86	0.88	Lu	0.57	0.82	0.94

**Table 2:** Physical properties of three coal ash samples.

Sample	Density (g/cm^3^)	Specific Surface Area (m^2^/g)	LOI ^1^ (%)	D50 ^2^(μm)
CA1	2.48	1.087	0	20.88
CA2	2.28	1.187	4.11	17.65
CA3	2.48	1.968	3.95	42.54

^1^ LOI -loss on ignition at 815 °C; ^2^ D50: The median diameter of the particle size distribution.

**Table 3:** The EDS analysis of points in Figure 5.

**Point A**			**Point B**			**Point C**		
**Elem.**	**Wt.%**	**At. %**	**Elem.**	**Wt.%**	**At. %**	**Elem.**	**Wt.%**	**At. %**
C	11.07	18.81	C	6.68	13.14	C	14.34	20.72
O	45.47	58.00	O	37.88	55.93	O	56.19	60.95
Al	10.72	8.11	Mg	4.33	4.21	Na	0.23	0.18
Si	8.41	6.11	Al	6.75	5.91	Mg	0.20	0.14
Ca	0.28	0.14	Si	3.98	3.34	Al	12.96	8.33
Ti	0.63	0.27	Ca	1.81	1.07	Si	14.95	9.23
Fe	23.43	8.56	Ti	1.13	0.56	K	0.58	0.26
			Mn	0.86	0.37	Ca	0.15	0.06
			Fe	36.59	15.48	Ti	14.34	20.72
						Fe	0.40	0.13
**Point D**			**Point E**			**Point F**		
**Elem.**	**Wt.%**	**At. %**	**Elem.**	**Wt.%**	**At. %**	**Elem.**	**Wt.%**	**At. %**
C	17.68	31.82	C	4.28	8.10	C	2.28	6.44
O	31.78	42.93	O	42.80	60.74	O	21.39	45.36
Na	0.20	0.19	Mg	7.63	7.13	Mg	1.29	1.80
Mg	0.52	0.46	Al	4.80	4.04	Al	0.58	0.73
Al	4.83	3.87	Si	5.32	4.30	Si	0.32	0.38
Si	5.91	4.55	Ca	8.57	4.85	Ca	1.01	0.85
K	0.42	0.23	Ti	0.36	0.17	Mn	1.84	1.14
Ca	0.53	0.29	Mn	0.86	0.36	Fe K	71.29	43.30
Ti	13.91	6.28	Fe K	25.37	10.31			
Fe	24.22	9.38						

Elem.: Elements. Wt.: weight. At.: atomic.

**Table 4:** The EDS analysis of points in Figure 7.

Point A			Point B			Point C		
Elem.	Wt.%	At. %	Elem.	Wt.%	At. %	Elem.	Wt.%	At. %
O	58.81	72.65	O	54.36	69.95	O	36.06	62.82
Mg	0.20	0.16	Al	22.82	17.42	Na	0.82	1.00
Al	23.85	17.47	Si	10.36	7.60	Mg	0.57	0.66
Si	8.26	5.81	P	3.81	2.54	Al	8.64	8.93
P	1.56	1.00	K	0.34	0.18	Si	13.05	12.95
K	0.26	0.13	Ca	2.06	1.06	K	1.17	0.83
Ca	2.89	1.43	Ti	0.67	0.29	Ca	0.65	0.45
Ti	1.96	0.81	Fe	0.68	0.25	Ti	1.58	0.92
Fe	1.12	0.40	La	1.81	0.27	Fe	13.34	6.66
Ce	1.09	0.15	Ce	3.08	0.45	La	6.05	1.21
						Nd	4.69	0.91
